# MicroRNA in Diabetic Nephropathy: Renin Angiotensin, AGE/RAGE, and Oxidative Stress Pathway

**DOI:** 10.1155/2013/173783

**Published:** 2013-12-15

**Authors:** Shinji Hagiwara, Aaron McClelland, Phillip Kantharidis

**Affiliations:** ^1^JDRF Danielle Alberti Memorial Centre for Diabetes Complications, Diabetes Division, Baker IDI Heart and Diabetes Institute, 75 Commercial Road, Melbourne, VIC 3004, Australia; ^2^Division of Nephrology, Department of Internal Medicine, Faculty of Medicine, Juntendo University, Tokyo 113-8421, Japan

## Abstract

MicroRNAs (miRNA) are a novel class of small, noncoding RNA molecules that have gained the attention of many researchers in recent years due to their ability to posttranscriptionally regulate the expression of families of genes simultaneously. Their role in normal physiology and pathobiology is intriguing and their regulation in normal and disease states is fascinating. That the cells can return to a state of homeostasis when these small molecules are perturbed is truly remarkable given the multiple cellular targets of each miRNA and that many mRNAs are targeted by multiple miRNAs. Several reviews have covered aspects of miRNA function in biology and disease. Here, we review the role of miRNA in regulating the renin-angiotensin system, AGE/RAGE signalling, and under conditions of oxidative stress in the context of diabetic nephropathy.

## 1. Introduction

The World Health Organization states that ~347 million people, roughly 9.5% of the adult population, were suffering from diabetes in 2008 [1]. The incidence of diabetes is rapidly increasing with estimates suggesting that this number will almost double by 2030. Diabetes mellitus is a major cause of chronic kidney disease (CKD) worldwide and is associated with enhanced morbidity and mortality, in particular accelerated cardiovascular disease [2, 3].

**Figure 1:**
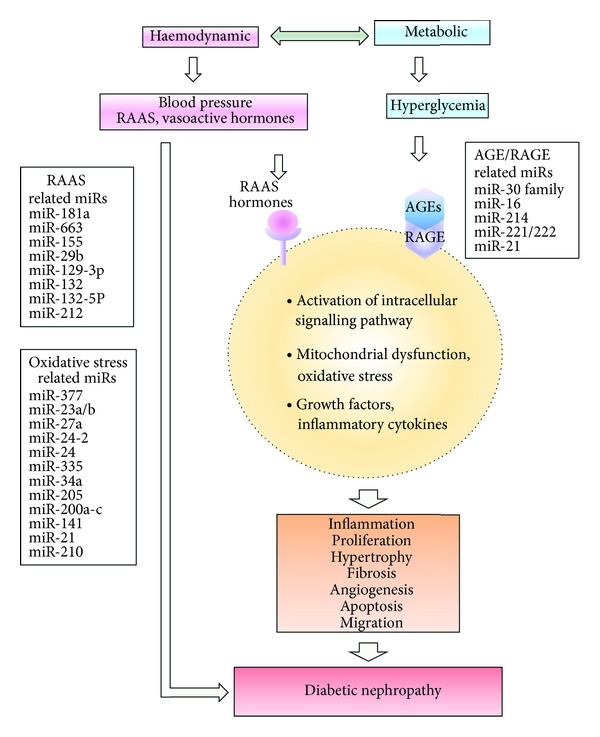
Schematic overview of mechanisms and microRNAs that are related to RAAS, AGE/RAGE, or oxidative stress contributing to diabetic nephropathy. AGE: advanced glycation end-product; miR: microRNA; RAAS: renin-angiotensin-aldosterone system; RAGE: receptor of AGE.

Diabetic nephropathy (DN) is now the most common cause of end-stage renal failure in the Western world [4]. Clinical associations that frequently precede overt DN are hypertension and poor glycaemic control [5], although a subset of patients develop nephropathy despite the proper glycemic control [6] and normal blood pressure. Once nephropathy is established, blood pressure often rises further, but glycaemic control can paradoxically improve as a result of reduced renal insulin clearance [7]. It is postulated that the interplay between metabolic and hemodynamic pathways plays an important role in the development and progression of DN [8] (Figure 1). Increased systemic and intraglomerular pressure is associated with increased albuminuria and glomerular injury. Activation of the renin-angiotensin-aldosterone system (RAAS) has been recognized as a key component of DN progression. Additionally, chronic hyperglycemia promotes the generation of advanced glycation end-products (AGEs). It is widely accepted that AGEs mediate their effects both directly and indirectly through receptor-dependent mechanisms. The receptor for AGE (RAGE) acts as a signal transduction receptor, and the RAGE-AGE interaction activates multiple intracellular signalling pathways which increase the production of growth factors, inflammatory cytokines, and oxidative stress (Figure 1).

In recent times, a novel class of non-coding RNA, microRNA (miRNA; miR), has been found to be expressed in all tissues and plays important roles in tissue homeostasis and disease progression [9, 10]. Whilst the role of miRNA in the pathogenesis of DN has been extensively reviewed by others in relation to growth factors and fibrosis in DN, the focus of this review is on the role of miRNA in the renin-angiotensin system and the AGE/RAGE signalling pathway, and their downstream the mediators such as oxidative stress and the immune response in the context of diabetic nephropathy.

## 2. Biogenesis and Function of miRNA

miRNAs are a group of small (~22 nucleotide) single-stranded non-coding RNAs ubiquitously expressed in plants and animals where they act posttranscriptionally to modulate the expression of target genes [11, 12]. They were first discovered in 1993 when lin-14 protein expression was found to be regulated by the mature product of the *lin-4* gene in *Caenorhabditis Elegans (C. Elegans) *[13]. The mechanism was found to rely upon sequence specificity in the 3′-untranslated region (UTR) of lin-14 to which lin-4 bound. Whilst this occurred with only partial complementarity, the binding occurred with sufficient affinity to result in inhibition of protein translation. At the time this was considered a peculiarity in the worm. It was not until 2000 when another such regulator, let-7, was discovered in *C. Elegans* [14], and soon after came the realisation that this form of regulation was conserved across many species, representing a general mechanism for regulating the expression of several genes.

miRNAs are found in intergenic sequences or on the antisense strand of genes and may possess their own promoter and regulatory sequences [15, 16]. Other miRNAs (almost 50% in the case of human miRNA) are found within gene sequences and are together regulated with their host gene [17–19]. Approximately half are found in polycistronic units from which the mature miRNAs are processed [16]. The primary transcript (pri-miRNA) is processed by a number of proteins including ribonuclease III, Drosha, and the RNA binding protein, DiGeorge syndrome critical region gene 8 (DGCR-8) protein, into a short hairpin RNA molecule termed the precursor miRNA (pre-miRNA) which is subsequently exported from the nucleus by exportin-5 [20–22]. Once in the cytosol, further processing is mediated by another ribonuclease III, Dicer, and this is followed by the incorporation of the mature strand of the duplex miRNA into the RNA-induced silencing complex (RISC) which includes the Argonaute family of proteins [23]. The miRNA-RISC complex stabilises the miRNA against nuclease attack and miRNA direct the complex to target RNA transcripts via sequence complimentarity between the miRNA seed sequence (2–8 nucleotides) and the miRNA recognition element (MRE) in the target transcript [20]. miRNAs modulate protein synthesis by binding to the 3′ UTR of mRNAs via incomplete base pairing [24] to the complementary seed sequence in the UTR, leading to translational repression. MREs are predominately located in the 3′ UTRs of mammalian mRNA, however; there is evidence indicating that miRNA can also mediate translational repression via binding 5′ UTRs or coding sequences [25, 26].

In plants and lower vertebrates where complete complimentarity exists between an miRNA seed sequence and the target mRNA, degradation of the target mRNA is induced. In contrast, miRNAs exercise finer control on protein expression in mammalian cells where they repress protein translation [27, 28], allowing for more comprehensive regulation of protein expression as any single miRNA can target many mRNAs, and any mRNA can be the target of several miRNAs [27]. This potential of miRNAs to regulate many genes adds significant complexity to the interpretation of many studies where the focus is on single genes. It is therefore critical to computationally detect the combination of miRNAs that target specific mRNA molecules. Such analyses often demand complex algorithms that need to be defined by high stringency levels to generate computational data that could then be assessed and validated in the wet lab. Added to this is the recent discovery of circulating miRNAs in microparticles [29] and the potential of these miRNAs to be delivered to sites distal to their generation.

## 3. miRNA and the Renin-Angiotensin System in Kidney Disease

Blood pressure is regulated by the renin-angiotensin-aldosterone system. Renin production is the key regulatory step that initiates an enzymatic cascade that leads to angiotensin generation and the control of blood pressure in addition to fluid and electrolyte homeostasis [30]. Renin is synthesized and released by renal juxtaglomerular (JG) cells, which are located at the entrance to the glomerulus. In early embryonic development, renin producing cells are broadly distributed along intrarenal arteries in the glomerular mesangium and in a few developing tubules. With maturation the cells become progressively restricted to the afferent arterioles. Lineage studies demonstrate that this progressive restriction is achieved by differentiation of renin cells into vascular smooth muscle cells (VSMCs), mesangial cells, and a subset of tubular cells [31].

Sequeira-Lopez et al. were the first to address the role of miRNA in the maintenance of JG cells by generating mice with a conditional deletion of Dicer (Dicer cKO mice) specifically and exclusively in renin-expressing cells, resulting in selective ablation of miRNA generation only in renin cells and their descendants [32]. Deletion of Dicer resulted in severe reduction in the number of JG cells accompanied by decreased expression of the renin *Ren1* and *Ren2* genes, decreased plasma renin concentration (PRC), decreased BP, abnormal renal function, and striking nephrovascular abnormalities including striped corticomedullary fibrosis. This study clearly showed a critical role of miRNA in the orchestration of renal function and the importance of miRNA homeostasis for specific organ functions.

The kidney has long been invoked in the etiology of essential hypertension. This could involve alterations in expression of specific genes and miRNA. Marques and colleagues conducted for the first time transcriptome-wide study of differential expression of mRNAs and miRNA in kidneys of hypertensive subjects (15 untreated hypertensive and 7 normotensive white males) by microarray technology [33]. They confirmed differences of expression for nuclear receptor subfamily 4 group A member 1 (NR4A1), NR4A2, NR4A3, period circadian protein homolog 1 (PER1), and salt-inducible kinase 1 (SIK1) mRNAs and for the miRNAs hsa-miR-638 and hsa-let-7c by real-time quantitative PCR expression in the medulla. Functional experiments confirmed the predicted binding of hsa-let-7c to the 3′ untranslated region of NR4A2 mRNA. In the renal cortex they confirmed differences in expression of apoptosis-inducing factor, mitochondrion-associated, 1 (AIFM1), alpha-1-microglobulin/bikunin precursor (AMBP), apolipoprotein E (APOE), cluster of differentiation 36 (CD36), ephrin-B1 (EFNB1), NADH dehydrogenase (ubiquinone) complex I, assembly factor 1 (NDUFAF1), peroxiredoxin 5 (PRDX5), REN, renin binding protein (RENBP), solute carrier family 13 (sodium/sulfate symporters), member 1 (SLC13A1), syntaxin 4 (STX4), and troponin T type 2 (cardiac) (TNNT2) mRNAs, and the miRNAs hsa-miR-21, hsa-miR-126, hsa-miR-181a, hsa-miR-196a, hsa-miR-451, hsa-miR-638, and hsa-miR-663. Functional experiments demonstrated that hsa-miR-663 can bind to the REN and APOE 3′ untranslated regions regulating REN and APOE mRNA levels, whereas hsa-miR-181a regulated REN and AIFM1 mRNA. A major discovery was evidence for REN mRNA regulation via binding of miRNAs hsa-miR-181a and hsa-miR-663 to its 3′ UTR as the observed downregulation of these 2 miRNAs in hypertension could explain the elevation in intrarenal renin mRNA. Due to small sampling size, these findings need to be bolstered by the acquisition of suitable samples from larger cohorts of untreated hypertensive subjects in other settings.

Chronic kidney disease (CKD) is a known cardiovascular risk factor, and most patients with CKD die of cardiovascular disease (CVD) before reaching the need for dialysis [37]. Chen and colleagues measured vascular miRNAs in blood from 90 patients with CKD and found an association between decreased circulating levels and progressive loss of eGFR by multivariate analyses [34]. Expression of vascular miRNAs was decreased in the thoracic aorta of CKD rats compared to normal rats, with concordant changes in target genes of RUNX2, AT1R, and myocardin with no alteration in DROSHA or DICER, indicating that the low levels of expression are not due to altered intracellular processing. Furthermore, the expression of miR-155 was negatively correlated with calcification of the aorta, a process known to be preceded by vascular dedifferentiation in these animals. Overexpression of miR-155 in VSMC from CKD rats inhibited AT1R expression and decreased cellular proliferation, confirming a causative role of low miR155 in VSMC transformation to a more synthetic, proliferative phenotype. However, whether downregulation of these miRNAs is the cause or consequence of the widespread vascular phenotype abnormalities in patients with CKD remains to be determined.

Jeppesen and colleagues have shown that AngII regulates five miRNAs (miR-29b, -129-3p, -132, -132-5P, and -212) during *in vitro* stimulation of primary cardiac fibroblasts and of HEK293N cells overexpressing the AT1R [35]. Furthermore, Eskildsen and colleagues undertook a detailed analysis of potential miRNAs involved in AngII-mediated hypertension in rats and hypertensive patients, using miRNA microarray and qPCR analysis. miR-132 and miR-212 are highly increased in the heart, aortic wall, and kidneys of rats with hypertension (159 ± 12 mm Hg) and cardiac hypertrophy following chronic AngII infusion. In addition, activation of the endothelin receptor, another G*α*q coupled receptor, also increased miR-132 and miR-212 [36]. A significant downregulation of miR-132 as well as a robust attenuation of miR-212 in human arteries from the ARB-treated patients was also observed, whereas treatment with *β*-blockers had no effect. In conclusion, miR-132 and miR-212 are upregulated in AngII-induced hypertension in organs associated with blood pressure control, possibly via the G*α*q-dependent pathway.

Table 1 contains miRNAs that are known to be regulated by the renin-angiotensin system in the kidney and other tissues and associated with kidney disease, hypertension, and cardiovascular disease.

## 4. miRNAs in AGE/RAGE and Kidney Disease

Chronic hyperglycemia promotes the generation of advanced glycation end products (AGEs) as a result of sequential biochemical reactions involving nonenzymatic glycation of protein and lipids known as the Maillard reaction [8]. As a consequence of AGE formation, there is often concomitant liberation of reactive oxygen species (ROS) [38]. AGEs can induce expression of the MCP-1 in podocytes through activation of the AGE receptor (RAGE) and generation of intracellular reactive oxygen species (ROS) [39]. The induction of oxidative stress results in upregulation of nuclear factor (NF)-*κ*B and various NF-*κ*B-mediated proinflammatory genes, eventually leading to glomerular and tubulointerstitial injury. Therefore, AGE/RAGE and oxidative stress signalling are important in the progression of DN and targeting this axis by modulating the miRNAs that are involved is a potential new therapy.

There are very few studies examining the role of miRNA in AGE/RAGE signalling related to kidney disease. Most studies have focussed on the role of RAGE in the immune system or cancer because RAGE is a member of the immunoglobulin superfamily of cell surface molecules. When Dicer was selectively inactivated in mouse podocytes, multiple abnormalities were observed in glomeruli of mutant mice, including foot process effacement, irregular and split areas of the glomerular basement membrane, podocyte apoptosis and depletion, mesangial expansion, capillary dilation, and glomerulosclerosis [40]. Gene profiling by microarray analyses revealed upregulation of 190 genes in glomeruli isolated from mutant mice at the onset of proteinuria compared with control littermates. Target sequences for 16 miRNAs were significantly enriched in the 3′-untranslated regions of the 190 upregulated genes. Further, supporting the validity of the *in silico* analysis, 6 of the 8 top-candidate miRNAs were identified in miRNA libraries generated from podocyte cultures; these included miR-28, miR-34a, and four members of the miR-30 family, miR-30c-1, miR-30b, miR-30d, and miR-30c-2. Among the 15 upregulated target genes of the miR-30 miRNA family, RAGE, vimentin, heat-shock protein 20, and immediate early response 3 were known to be expressed in injured podocytes in experimental models and human kidney disease. RAGE and immediate early response 3 are known to mediate podocyte apoptosis, whereas vimentin and heat-shock protein 20 are involved in cytoskeletal structure [40]. The findings demonstrate the important roles of the mir-30 family in podocyte homeostasis and podocytopathies.

AGE/RAGE interaction induces inflammatory genes such as cyclooxygenase-2 (COX-2) [41]. S100b, a RAGE ligand, significantly increased COX-2 mRNA accumulation in THP-1 monocytes at 2 h via mRNA stability. S100b decreased occupancy of the DNA/RNA-binding protein, heterogeneous nuclear ribonuclear protein K (hnRNPK), at the COX-2 promoter but simultaneously increased its binding to the COX-2 3′-UTR. Additionally, S100b significantly downregulated the expression miR-16 which acts to destabilize COX-2 mRNA by binding to its 3′-UTR. hnRNPK knockdown increased miR-16 binding to COX-2 3′-UTR indicating a crosstalk between them. These results demonstrate that diabetic stimuli can efficiently stabilize inflammatory genes via opposing actions of key RNA-binding proteins and miRNA [41].

AGEs delay spontaneous apoptosis of monocytes and contribute to the development of inflammatory responses [42]. In genome-wide miRNA expression analysis significant upregulation of miR-214 was consistently observed in THP-1 and human monocytes treated with various AGEs. A striking increase in miR-214 was also detected in monocytes from patients with chronic renal failure. PTEN was identified as a target gene of miR-214. Overexpression of pre-miR-214 led to impaired PTEN expression and delayed apoptosis of THP-1 cells, whereas knockdown of miR-214 levels largely abolished AGE-induced cell survival. These findings define a role for miR-214-targeting of PTEN in AGE-induced monocyte survival [42].

High-mobility group box 1 protein (HMGB1) is a late inflammatory cytokine that signals danger to the immune system through the RAGE and Toll-like receptor [43]. miR-221 and miR-222 are involved in cell proliferation through the inhibition of the cell cycle regulator, p27kip1, in smooth muscle cells [44] and endothelial cells (ECs) [45]. HMGB1 increases the expression of miR-221 and miR-222 in primary cultures of excised papillary lesions and in an established papillary cancer cell line and overexpression of miR-222 and miR-221 caused by HMGB1 increases growth and motility in papillary thyroid cancer cells. Recently, it was reported that miR-21 and miR-221, which are tissue inhibitors of metalloproteinases (TIMP)3-targeting miRNA, were significantly upregulated in kidneys from diabetic mice compared to control littermates and in a mesangial cell line grown in high glucose conditions [46]. Mesangial expansion is one of the main characters in DN and is mainly due to accumulation of extracellular matrix (ECM). ECM turnover is regulated by TIMPs activities. In diabetic conditions, TIMP3 expression in kidney is strongly reduced, but the causes of this reduction are still unknown. miR-221/miR-222 cluster which has been examined in several cardiovascular disorders affects the angiogenic activity of stem cell factor (SCF) by targeting the receptor c-Kit [47]. High glucose levels elevate miR-221 and this correlates with decreased expression of c-kit and reduced EC migration [48]. miR-221/222 is also considered to be important in atherosclerosis. miR-221/222 targets NO synthase and transcription factor ETS-1 which are both major contributors to the atherosclerotic process. [47, 49, 50] Taken together, these results indicate that HMGB1/RAGE and miR-221/222 axis may be activated in the kidney and vasculature resulting in ECM accumulation and atherosclerosis in DN patients.

Table 2 lists the miRNAs that are known to be modulated by the AGE/RAGE in kidney, monocytes, VSMCs, and ECs and associated with kidney disease and vascular dysfunction.

## 5. miRNAs in Oxidative Stress and Kidney Disease

Reactive oxygen species (ROS) is a collective term that includes a number of reactive and partially reduced oxygen (O_2_) metabolites. Some of them are free radicals, such as superoxide anion (O_2_^−^) and hydroxyl radicals (^•^OH) that are extremely reactive molecular species with an unpaired electron in their outer orbital. A number of studies have demonstrated that both type 1 and type 2 diabetes are associated with overproduction of oxygen-derived free radicals. Increased ROS production and reduced levels of antioxidants culminate with an in increased level of oxidative stress leading to oxidative damage to cellular components [51]. Among the many enzymatic systems implicated in ROS generation in vascular tissues, enzymes of the mitochondrial respiratory chain (complexes I and III), xanthine oxidase, uncoupled nitric oxide synthase, and nicotinamide adenine dinucleotide phosphate reduced form (NADPH) oxidase (NOX) appear to be particularly important [52–54]. Increased NOX-mediated superoxide production has been reported in experimental models of diabetes and occurs in parallel with upregulation of NOX1 and NOX4 [55, 56]. NOX-mediated generation of superoxide is an important mediator of matrix accumulation, renal fibrosis, and podocyte injury in DN [57].

miR-377 is upregulated in spontaneous and STZ-induced mouse models of DN and in mesangial cells exposed to high glucose and TGF-*β*1 [58]. Stable overexpression of miR-377 in mesangial cells increased fibronectin synthesis. Furthermore, genes potentially relevant to the pathogenesis of DN were confirmed experimentally, including the cytoskeletal regulator p21-activated kinase 1 (PAK1) and superoxide dismutases (SOD1/SOD2) that catalyse ROS, which accumulates in response to hyperglycemia [58].

It has previously been shown that upregulation of the miR-23a~27a~24-2 cluster induces caspase-dependent and caspase-independent cell death in human embryonic kidney cells via c-jun N-terminal kinase (JNK) with increases in ROS and the release of proapoptotic factors such as cytochrome *c* (cyt c) in addition to apoptosis-inducing factor (AIF) from the intermembrane space of mitochondria to the cytosol [59]. In order to better understand the molecular mechanism responsible for miR-23a~27a~24-2 cluster-induced cell death, gene expression profiling was performed in control and miR-23a~27a~24-2 cluster overexpressing HEK293T cells. This revealed miR-23a~27a~24-2 cluster-induced apoptosis was associated with endoplasmic reticulum (ER) stress and the unfolded protein response (UPR) pathways in HEK293T cells. Overexpression of the miR-23a~27a~24-2 cluster resulted in ER stress and altered mitochondrial membrane permeability and this was further established by increased intracellular and mitochondrial calcium levels in HEK293T cells [60].

There have been reports that miR-23b levels were increased in the kidneys of TGF-*β*1 transgenic mice and rats following subtotal nephrectomy, which was found to be localized to podocytes and tubular epithelium by *in situ* hybridization. miR-23b was also upregulated by TGF-*β*1 in cultured renal epithelial cells. *In vitro* gain and loss-of-function studies pointed to several miR-23b targets, including TGF-*β* receptor type II, SMAD3, and TGF-*β*1 itself, suggesting a negative feedback loop-regulating TGF-*β*1 signaling. Modulation of miR-23b in cultured podocytes altered expression of WT1, nephrin, and podocin and also influenced motility of cultured tubular cells [61].

Studies have found that in the aged organs, including the kidney, expression of antioxidant enzymes such as SOD, catalase, Gpx, and peroxiredoxins is downregulated [62, 63] thus leading to reduced antioxidant capacity. From bioinformatic analysis of the miRNA expression profile of young and old rat kidneys, mitochondrial SOD2 and thioredoxin reductase 2 (Txnrd2) are potential targets of miR-335 and miR-34a, respectively, as aging mesangial cells exhibited significant upregulation of miR-335 and miR-34a and marked downregulation of SOD2 and Txnrd2. Further studies confirmed SOD2 and Txnrd2 as target genes of miR-335 and miR-34a which coincided with ROS generation [64].

Within the mitochondria, uncoupling protein 2 (UCP2) has recently been reported as a negative regulator of ROS generation [65]. Its ablation leads to marked increase of oxidative stress in several cell types [66]. In the stroke-prone spontaneously hypertensive rat (SHRsp) kidneys, severe renal damage along with increased rate of inflammation and oxidative stress was observed [67]. *UCP2* gene and protein levels were downregulated paralleled by differential expression of kidney miR-24 and -34a, which were identified to target the *UCP2* gene. The silencing of the *UCP2* gene in renal mesangial cells led to increased rate of ROS generation, increased inflammation and apoptosis, reduced cell vitality, and increased necrosis, suggesting that UCP2 is critical in preventing oxidative stress damage in renal mesangial cells [67].

Specific miRNAs, including miR-205 and the miR-200 family (miR-200a–c, miR-141, and miR-429), were shown to mediate epithelial-to-mesenchymal transition (EMT) in response to TGF-*β*1 in Madin-Darby canine kidney cells [68]. EMT is thought to be an important event driving renal fibrosis. Downregulation of these miRNAs relieves their cooperative repression of the mesenchymal transcription factors ZEB1 and SIP1 that, in turn, are free to inhibit E-cadherin expression promoting epithelial dedifferentiation.

Muratsu-Ikeda et al. assessed changes in miRNA expression in the cultured renal tubular cell line HK-2 under hypoxia-reoxygenation-induced oxidative stress or ER stress using miRNA microarray assay and real-time RT-PCR. Among altered miRNA expression, miR-205 was markedly decreased in both stress conditions. Functional analysis revealed that decreased miR-205 led to an increase in cell susceptibility to oxidative and ER stresses and that this increase was associated with the induction of intracellular ROS and suppression of antioxidant enzymes. Furthermore, miR-205 bound to the 3′-UTR of the prolyl hydroxylase 1 (PHD1/EGLN2) gene and suppressed the transcription level of EGLN2, which modulates both intracellular ROS level and ER stress state [69].

miRNA profiling of HUVEC treated for 8 and 24 hrs with 200 *μ*M hydrogen peroxide (H_2_O_2)_ showed that miR-200c and the cotranscribed miR-141 increased more than eightfold. The other miR-200 family members were also induced, albeit at a lower level [70]. miR-200c overexpression in HUVEC recapitulates many aspects of the oxidative stress-induced phenotype, since it induces cell growth arrest, apoptosis, and cellular senescence. All these effects are mediated, at least in part, by the inhibition of the target ZEB1 by the miR-200 family [70]. miR-200 family induction following H_2_O_2_ exposure has been confirmed in different cell lines such as human and mouse immortalized fibroblasts, colon carcinoma (CT26), mammary gland epithelial cells (NMuMG) and human cell lines, melanoma cells (MDA-MB-435S), kidney cells (293T), breast adenocarcinoma (MDA-MB-436 and BT-549), and ovarian adenocarcinoma (SKOV3). Notably, in all these cell lines, all miR-200 family members were upregulated [71]. In a recent study, an analysis of miRNAs upregulated in diabetic mouse heart compared to control was performed revealing that miR-200c and miR-141 were among the most upregulated [72]. The authors show that miR-141 targets the inner mitochondrial membrane phosphate transporter, solute carrier family 25 member 3 (Slc25a3), which provides inorganic phosphate to the mitochondrial matrix and is essential for ATP production, suggesting an important role of miR-200 family in mitochondrial responses involved in cardiac diseases associated with diabetes and obesity.

On the contrary to the role for miR-200 family in oxidative stress, miR-200 family has been reported for antifibrotic roles in DN. The miR-200 family also plays an important role in EMT, which is considered to mediate production of renal fibroblasts, in part by targeting ZEB1/2, the transcriptional repressors of E-cad [68, 77, 78]. On the other hand, another group has demonstrated that TGF-*β* activated Akt in glomerular mesangial cells by inducing miR-200b and miR-200c, both of which target FOG2, an inhibitor of phosphatidylinositol 3-kinase activation [79], suggesting the role of miR-200 family on glomerular mesangial hypertrophy in the progression of DN.

The contrasting findings above highlight the complex nature of miRNA research. Some of the differences may relate to variances in experimental models and/or conditions; however, one often overlooked explanation is that some effects of miRNA and inhibitors are likely to be indirect in nature. Our understanding of miRNA function is continually evolving. Recent evidence demonstrates regulation of gene expression via deadenylation, by altering message stability, and by effects on transcription. Bioinformatics utilising pathway analysis will be needed to better understand the crosstalk between factors that drive many downstream processes and how those processes ultimately impact the expression of individual genes.

miRNA profiling of rat VSMCs treated with 200 *μ*M H_2_O_2_ for 6 hours revealed an upregulation of miR-21 [73]. This study showed that miR-21 participates in H_2_O_2_-mediated gene regulation via its target programmed cell death 4 (PDCD4) and transcription factor AP-1 pathway activity. Elevated miR-21 is thought to contribute to atherosclerosis by directly targeting PPAR*α*, leading to an increased inflammatory response in ECs. Inhibition of miR-21 causes activation of AP-1 as well as upregulation of proinflammatory factors such as VCAM-1 and MCP-1 [80]. miR-21 can also act as an inhibitor of angiogenesis by reducing EC proliferation, migration, and tube formation in culture via inhibition of RhoB. miR-21 can also increase nitric oxide (NO) in HUVECs exposed to shear stress via increased phosphorylation of nitric oxide synthase (NOS) and decreased apoptosis [74]. This activity is balanced by the ability of miR-21 to repress SOD-2 which is important in antioxidant defence. Furthermore, miR-21 is elevated in angiogenic precursor cells by asymmetrical dimethylarginine (ADMA) which is a powerful NOS inhibitor, ultimately resulting in elevated intracellular reactive oxygen (ROS) species [75]. Increased miR-21 levels were recently demonstrated in renal cortex in db/db mice [81]. Knockdown of miR-21 decreased mesangial expansion, collagen I/IV, and FN1 expression and reduced macrophage infiltration and TNF*α* and MCP-1 expression. The gene expression changes were replicated *in vitro* in both PTC and mesangial cells (MCs) with miR-21 overexpression enhancing fibrogenesis via a mechanism which in part involved the direct targeting of SMAD7. Interestingly, miR-21 targets PTEN and also leads to decreased mesangial expansion in *db/db* mice [82]. miR-21 has also been implicated in regulation of TGF-*β* signalling in a number of animal models of tubulointerstitial fibrosis and associated renal dysfunction. In one such model, SMAD7 overexpression in the rat unilateral ureteral obstruction model has restored miR-21 expression to normal levels with congruent improvements in renal pathology [83]. In line with a profibrotic role for miR-21, upregulation of this miRNA is inhibited by SMAD3 deletion in an obstructive nephropathy model [84]. Furthermore, regulation of PDCD4 by miR-21 enhances podocyte apoptosis and loss in conjunction with increased tubular epithelial cells survival against growth arrest signals [85, 86].

miR-210 appears to function as master regulator of the hypoxic response as it was found to be upregulated by hypoxia in virtually all the cell types tested to date [87, 88]. Recent data demonstrate that Hif1*α* can block both mitochondrial respiration via the electron transport chain (ETC) through transcriptional activation of miR-210 in some cell types [87, 88]. Upregulation of miR-210 along with VEGF and VEGFR2 expression was confirmed in renal ischemia/reperfusion (I/R) injury of male Balb/c mice. Furthermore, overexpression of miR-210 in HUVEC-12 cells enhanced VEGF and VEGFR2 expression and promoted angiogenesis in matrigel *in vitro*. These results suggest that miR-210 may be involved in targeting the VEGF signaling pathway to regulate angiogenesis after renal I/R injury [76].

Table 3 summarises the miRNAs that are regulated by oxidative stress in the kidney and other tissues and associated with kidney disease and vascular dysfunction resulting from excessive ROS production.

## 6. Future Perspectives

Considerable progress has been made in identifying a number of important roles for miRNA in various biological processes and in disease. There is much excitement at the prospect that some miRNAs appear to be important to the regulation of several related processes in diabetes and its complications, including the modulation of the RAAS and oxidative stress pathways. The number of miRNAs relevant to these conditions is constantly increasing (Tables 1–3). It is encouraging that in some cases restoring the expression of a dysregulated miRNA can attenuate or even reverse disease. Our initial understanding of gene regulation has continued to change from simple concepts in terms of protein factors sitting on DNA to complex epigenetics involving chromatin dynamics and multiple histone and DNA modifications. Even this complexity has been superseded by the ability of miRNA to modulate the expression of multiple targets posttranscriptionally. Whilst the biology around miRNA continues to generate new and interesting findings, the challenge of the future is to translate some of the exciting experimental findings to the potential therapeutic interventions.

## Figures and Tables

**Table 1:** Relevant miRNA in RAAS.

miRNAs	Tissue/organ/cell line	Source	Target	Functions	Reference
miR-181a	Kidney	Hypertensive patients	REN, AIFM1	Hypertension	[33]
miR-663			REN, APOE		

miR-155	Blood	CKD patients	AT1R	Hypertension, cardiovascular disease	[34]

miR-29b, -129-3p, -132, -132-5P and -212	HEK293N cells, cardiac fibroblasts	Human, Rat		Associated with cardiovascular disease	[35]

miR-132/212	Heart, aortic wall, and kidneys	Rat		Associated with blood pressure control	[36]
	Artery	ARB-treated patients			

AIFM1: apoptosis-inducing factor, mitochondrion-associated, 1; APOE: apolipoprotein E; AT1R: angiotensin II receptor, type 1; CKD: chronic kidney disease; HEK: human embryonic kidney cells; RAAS: renin-angiotensin-aldosterone system; REN: renin.

**Table 2:** Relevant miRNA in AGE/RAGE.

miRNAs	Tissue/organ/cell line	Source	Target	Functions	Reference
miR-30 family	Podocyte	Mice	AGER, Vim, HSP20, Ier3	Podocyte homeostasis and podocytopathies	[40]

miR-16	THP-1 monocytic cells	Human	COX-2	Regulate inflammation	[41]

miR-214	THP-1 monocytic cells	Human	PTEN	Monocyte survival	[42]

miR-221/222	VSMCs	Rat	p27^Kip1^ and p57^Kip2^	Cell proliferation	[44]

miR-221/222	Endothelial cells	Human	p27^Kip1^ and p57^Kip2^	Cell proliferation	[45]

miR-21 and miR-221	MES 13 mesangial cells	Mice	Timp3	DN progression	[46]
miR-21	Kidney	Human			

miR-221/miR-222	HUVECs	Human	c-Kit	Reduced EC migration	[48]

AGE: advanced glycation end-product; AGER: advanced glycosylation end-product-specific receptor; c-kit: V-kit Hardy-Zuckerman 4 feline sarcoma viral oncogene homolog; COX: Cyclooxygenase; DN: diabetic nephropathy; EC: endothelial cells; HSP: Heat shock protein; HUVECs: human umbilical vein endothelial cells; Ier3: immediate early response 3; p27^Kip1^: Cyclin-Dependent Kinase Inhibitor 1B; p57^Kip2^: Cyclin-Dependent Kinase Inhibitor 1C; PTEN: phosphatase and tensin homolog; RAGE: Receptor for AGE; Timp: metallopeptidase inhibitor; Vim: Vimentin; VSMCs: vascular smooth muscle cells.

**Table 3:** Relevant miRNA in oxidative stress.

miRNAs	Tissue/organ/cell line	Source	Target	Functions	Reference
	NHMC	Human	SOD1/2, PAK1	Fibronectin synthesis	[58]
miR-377	MES 13 mesangial cells	Mouse			
	Kidney	Mouse			

miR-23a~27a~24-2	HEK293T	Human		ER stress and UPR pathways-associated apoptosis	[60]

miR-23b	Renal epithelial cells, podocyte	Mice, Rat	TGF-*β* receptor type II, SMAD3, TGF-*β*1	Negative feedback loop-regulating TGF-*β*1 signaling	[61]

miR-335	Kidney, primary mesangial cells	Rat	SOD2	Renal aging	[64]
miR-34a			Txnrd2		

miR-24 miR-34a	Kidney, CRL 2573 mesangial cell	Rat	UCP2	Oxidative stress damage	[67]

miR-205	HK-2 renal tubular cell	Human	PHD1/EGLN2	Antioxidative and anti-ER stress	[69]

miR-200c	HUVEC	Human	ZEB1	Cell growth arrest, apoptosis, and cellular senescence	[70]

miR-200a/-141	Fibroblasts,	Mice	p38*α*	Enhanced oxidative stress, tumorigenesis, and chemosensitivity	[71]
	CT26 colon carcinoma,	Mice			
	NMuMG mammary gland epithelial cells,	Mice			
	MDA-MB-435S melanoma cells,	Human			
	293T kidney cells,	Human			
	MDA-MB-436 and BT-549 breast adenocarcinoma,	Human			
	SKOV3 ovarian adenocarcinoma	Human			

miR-200c/-141	Heart	Mice	Slc25a3	Decreased mitochondrial ATP production	[72]
	HEK293	Human			

miR-21	VSMCs	Rat	PDCD4	Cellular injury	[73]

miR-21	HUVECs	Human	PTEN	Increased NO production, reduced apoptosis	[74]

miR-21	Angiogenic progenitor cell	Human	SOD2	Increased intracellular ROS concentration and impaired NO bioavailability	[75]

miR-210	Kidney	Mice		Activation of VEGF Signaling pathway	[76]
	HUVECs	Human			

ATP: adenosine-triphosphate; ER: endoplasmic reticulum; HEK: human embryonic kidney cells; HUVECs: human umbilical vein endothelial cells; NF-kB: nuclear factor k-light-chain-enhancer of activated B cells; NHMC: normal human mesangial cells; NO: nitric oxide; PAK1: P21 protein (Cdc42/Rac)-activated kinase 1; PDCD4: programmed cell death 4; PHD1/EGLN2: prolyl hydroxylase 1; PTEN: phosphatase and tensin homolog; ROS: reactive oxygen; Slc25a3: solute carrier family 25 member 3; SOD: superoxide dismutase; TGF-*β*: transforming growth factor-*β*; Txnrd2: thioredoxin reductase 2; UCP2: uncoupling protein 2; UPR: unfolded protein response; VEGF: vascular endothelial growth factor; VEGF: vascular endothelial growth factor; ZEB1: zinc finger E-box-binding homeobox 1.
